# Comorbidity and household income as mediators of gender inequalities in dementia risk: a real-world data population study

**DOI:** 10.1186/s12877-024-04770-3

**Published:** 2024-02-29

**Authors:** Uxue Zubiagirre, Oliver Ibarrondo, Igor Larrañaga, Myriam Soto-Gordoa, Lorea Mar-Barrutia, Javier Mar

**Affiliations:** 1 Biodonostia Health Research Institute, Donostia-San Sebastián, Guipúzcoa, Spain; 2 Basque Health Service (Osakidetza), Debagoiena Integrated Healthcare Organisation, Research Unit, Arrasate-Mondragón, Guipúzcoa, Spain; 3 Kronikgune Institute for Health Service Research, Barakaldo, Spain; 4 Faculty of Engineering, Electronics and Computing Department, Mondragon Unibertsitatea, Mondragon, Gipuzkoa Spain; 5 Department of Psychiatry, Osakidetza Basque Health Service, Araba University Hospital, Vitoria- Gasteiz, Spain

**Keywords:** Dementia, Income level, Gender, Comorbidity, Incidence, Prevalence, Mediation, Real-world data, Regression

## Abstract

**Background:**

Low household income (HI), comorbidities and female sex are associated with an increased risk of dementia. The aim of this study was to measure the mediating effect of comorbidity and HI on the excess risk due to gender in relation to the incidence and prevalence of dementia in the general population.

**Methods:**

A retrospective and observational study using real-world data analysed all people over 60 who were registered with the Basque Health Service in Gipuzkoa. The study measured HI level, the Charlson comorbidity index (CCI), age and sex. The prevalence and incidence of dementia were analysed using logistic regression and Poisson regression models, respectively, adjusted by HI, sex, comorbidity and age. We estimated the combined mediation effect of HI and comorbidity on the prevalence of dementia associated with gender.

**Results:**

Of the 221,777 individuals, 3.85% (8,549) had a diagnosis of dementia as of 31 December 2021. Classification by the CCI showed a gradient with 2.90% in CCI 0–1, 10.60% in CCI 2–3 and 18.01% in CCI > 3. Both low HI and gender were associated with a higher crude prevalence of dementia. However, in the CCI-adjusted model, women had an increased risk of dementia, while HI was no longer statistically significant. The incidence analysis produced similar results, although HI was not significant in any model. The CCI was significantly higher for men and for people with low HI. The mediation was statistically significant, and the CCI and HI explained 79% of the gender effect.

**Conclusions:**

Comorbidity and low HI act as mediators in the increased risk of dementia associated with female sex. Given the difference in the prevalence of comorbidities by HI, individual interventions to control comorbidities could not only prevent dementia but also reduce inequalities, as the risk is greater in the most disadvantaged population.

**Supplementary Information:**

The online version contains supplementary material available at 10.1186/s12877-024-04770-3.

## Background

Dementia has been identified as a key public health challenge due to the rapid ageing of the population [1] and the consequences of dementia in terms of disability, reduced quality of life and institutionalisation [2, 3]. Given the lack of curative treatments on the horizon, prevention appears to be a feasible option [4]. An additional objective of prevention programmes should be the reduction of the large inequalities associated with gender and socioeconomic level [5, 6], which would be highly cost-effective [7]. Most studies indicate a higher prevalence of dementia in women [8–12]. In a systematic review, the higher prevalence of dementia in women was explained by gender differences in life expectancy and education [9]. However, causes of differences in the incidence between men and women are not clear [8, 13]. The evidence is overwhelming regarding the social gradient in the risk of dementia, with higher risk in the most disadvantaged population and in certain ethnic groups [5, 6, 9, 14]. Although the prevalence of genetic determinants is heterogeneous in different ethnic groups, social determinants account for the majority of the variance in health [14, 15]. In addition, the results suggest that comorbidity plays a role not only in vascular dementia but also in dementia associated with neurodegeneration and cognitive decline in general [8, 16, 17]. Thus, the analysis of inequalities in dementia requires the joint consideration of social determinants, gender and possible mediators, such as comorbidity [18]. The problem in this analysis lies in the heterogeneity of the variables included in different studies and in the correlation between them [19]. As the 2020 report of the Lancet Commission pointed out, knowledge about risk factors and potential prevention, detection, and diagnosis of dementia is improving although significant gaps remain [20].

Socioeconomic factors are not separate from other risk factors [21]; therefore, the prevention of dementia requires a better understanding of the relationship between the clinical determinants or comorbidities and individuals’ gender, age and household income [22]. In combination with gender and household income, comorbidities can be used to estimate the difference in risk associated with each variable [23]. However, no similar studies have examined the combined risk of dementia based on gender, socioeconomic determinants and comorbidities [24].

Real-world data (RWD) studies based on electronic health records are increasingly used to study the epidemiology of dementia [2, 25]. Their advantage is their large sample size and the ability to obtain representative population indicators in the case of Beveridge health systems [26]. Analysis of inequalities in the Spanish health system is possible because of the inclusion of the pharmaceutical copayment category in these databases, which provides an objective indicator of household income level [27, 28]. The availability of a record of all diagnoses for each individual facilitates the collection of comorbidity information for the entire population [23]. As a limitation, it has been noted that the use of RWD requires validation studies to determine the real content of what is being measured [29, 30]. In general, the availability of databases with complete country records and the publication of recommendations about their appropriate use has led to exponential growth in evidence based on RWD [31, 32].

Identification of the determinants of the development of dementia will help to define the priorities in prevention programmes according to each country’s social and health characteristics. The aim of this study was to measure the mediating effect of comorbidity and household income on gender-related risk for the recorded dementia incidence and prevalence in the general population.

## Methods

A retrospective and observational (real-world data) study was conducted to identify all primary care and hospital diagnoses of people over the age of 60 years registered in the database of the Basque Health Service in Gipuzkoa as of 1 January 2022 and 1 January 2021. Gipuzkoa is a province of Basque Country with a total population of 716,616 inhabitants. We relied on the Oracle Analytics Server (OAS) (Basque Country), which has anonymously stored administrative, laboratory, pharmaceutical and clinical data for all users of public health services in all primary care centres and hospitals (outpatient, emergency, and hospitalisation) since 2003. From this source, we directly obtained the variables necessary to identify dementia cases and all diagnoses included in the comorbidity score. Access to the Basque Health Service is universal for all residents based on a Beveridge model. However, 20% of the population has complementary private insurance. As private insurance does not reimburse pharmacy prescriptions, individuals with dual coverage make use of both healthcare delivery systems. Therefore, the OAS database shows a high representativeness of the Basque population. The study protocol was approved by the Clinical Research Ethics Committee (CEIC) of the Basque Country with registration number PI2021085.

In the Basque Health Service, access to healthcare requires the physician to assign a code based on the International Classification of Diseases (ICD), which enables the diagnosis to be coded in the record system. The ICD-9-CM was used until 2015, and the ICD-10 has been used since 2016. Individuals who experienced an event with a code associated with dementia in the ICD-9 (290**, 2941*, 331**) and ICD-10 (F015*, F028*, F039*, G300*, G301*, G308*, G309*, G310*, G311*, G312*, G318*, G319*) were identified. The diagnosis of dementia using this procedure has been shown to be adequate, with positive predictive values of 95.1% and negative predictive values of 99.4% [29].

The variables included in the study were age, sex, dementia diagnosis with date, household income based on pharmacy copayment and diagnoses included in the Charlson Comorbidity Index (CCI) that is a tool to standardise the comorbidities of individuals in a population [33, 34]. The CCI contains 19 diagnoses (acute myocardial infarction, congestive heart failure, peripheral vascular disease, stroke, pulmonary disease, connective tissue disorder, peptic ulcer, liver disease, diabetes, diabetes complications, paraplegia, kidney disease, cancer, metastatic cancer, severe liver disease, HIV and dementia) [34, 35]. Age was classified into the following groups: 60–64, 65–69, 70–74, 75–79, 80–84, 85–89, 90–94, and 95 and over. The CCI was classified into two categories, 0–1 and > = 2.

As a socioeconomic indicator, we used household income that determines individuals’ pharmacy copayment categories classified into a low-income and a high-income category [27, 28]. The low- household income group included people with disabilities, integration incomes, and noncontributory pensions and those without unemployment benefits (TSI code 001) as well as people with income (workers or pensioners) lower than 18,000 euros (TSI code 002 limit 01 and TSI code 003). High household income included people with income (workers or pensioners) higher than 18,000 euros and mutualists and passive classes (TSI codes 002 limit 02, TSI code 004, TSI code 005, TSI code 006).

Sex refers to the biological characteristics (primary and secondary) that differentiate females from males while gender refers to the socially determined meaning of being a man or a woman, which shapes the definition of feminine and masculine behaviours, products, technologies, environments and knowledge in a particular society [36]. Given that social functions help to explain the gender differences observed in health and that women systematically report poorer health than men, an integrated social and gender framework has been proposed to understand inequalities in health [37]. However, our dataset did not contain gender as “the socially constructed identity of individuals” [38]. Therefore, we used sex as a surrogate. We referred to gender in the introduction and the discussion to take into account the social and cultural dimension of gender, [36] but we used the term sex in the methods and results sections.

### Statistical analysis

We used R software (version 4.2.2) for statistical analysis and set the significance threshold at *p* < 0.05. After the two datasets (January 1, 2021 and 2022) were extracted from OAS, cases of dementia were identified. We also analysed whether cases from 2021 were still alive in 2022 and whether cases from 2022 were present in 2021. In this way, cases of dementia were classified into the following groups: alive in 2022 and diagnosis already present in 2021, alive in 2022 and diagnosis absent in 2021, deceased in 2022 and diagnosis already present in 2021, and deceased in 2022 and diagnosis absent in 2021. First, we conducted a descriptive analysis of the entire population and calculated the prevalence and incidence of dementia. The disaggregated prevalence of dementia by age on 1 January 2022 was calculated by dividing the number of cases by the total population. The numerator for the incidence of dementia included the cases identified in 2022 that were not present in the 2021 dataset. To calculate the rate per 1000 person-years, the population on 1 January 2021 was used as the denominator, and the observation years lost due to deaths and dementia cases identified in 2021 were subtracted. It was assumed that both events occurred uniformly throughout the year, so a duration of 0.5 years was assigned to them. The results were validated by comparing them with the literature [2]. Incidence was analysed via several Poisson models [39]. To fit the Poisson models, we used the Akaike information criteria (AIC). The basic idea of the AIC is to penalise the inclusion of additional variables in a model, so it adds a penalty that increases the error when including additional terms. The lower the AIC is, the better the model. We conducted a logistic regression model to analyse the role of different covariates (continuous CCI, age, and gender) to adjust the prevalence of dementia by household income. A linear regression estimated the risk of comorbidity measured by the CCI according to household income adjusted by gender and age.

To calculate the percentage of explanation of the risk of dementia associated with sex and mediated by the CCI and household income, mediation analyses with two variables were conducted using the regression-based approach with the CMAverse R package [40]. We performed logistic regressions in which the CCI was categorised into 0–1 and > = 2 [41, 42]. The first model included the risk of dementia as the outcome variable, sex as the independent variable, and age, household income and CCI as covariates. The binary CCI was the outcome variable of the second model that had household income and age as covariates. The third model had household income as the outcome variable and sex and age as covariates. In this way, we calculated the mediation effect of CCI and household income on the sex-associated dementia prevalence risk. This procedure rendered four indicators, Rte, Rtnde, Rtnie and Pm. Rtnde represented the total direct effect of sex on dementia without considering the role of CCI and household income as mediating variables. Rtnie indicated the total indirect effect of sex on dementia operating through the CCI or household income as mediator without considering a direct effect of sex on dementia. Rte was the total effect calculated as the product of Rtnde and Rpnie. Finally, Pm provided the percentage of the total relationship between the exposure variable (sex) and the response variable (dementia) that was explained by the mediator variables (low household income and CCI).

## Results

In Table 1, the sociodemographic (age, sex and socioeconomic level) and clinical (CCI) characteristics of the population over 60 with a diagnosis of dementia on 1 January 2022 are shown. Of the 221,777 individuals in the population, 8,549 (3.85%) had a diagnosis of dementia on 1 January 2021. Classification by CCI showed a gradient with 2.90% in the CCI 0–1 group, 10.60% in the CCI 2–3 group, and 18.01% in the CCI above 3 group. The prevalence was also higher in individuals with low household income (4.87%) and women (4.78%). As expected, the prevalence in groups older than 75 years exceeded the average, and the 90–95 age category showed the highest Fig. (14.76%).

**Table 1:** Sociodemographic and clinical characteristics of the population over 60 according to dementia diagnosis as of 1 January 2022 (prevalence)

	Dementia	%	No dementia	%
Total	8,549	3.85%	213,228	96.15%
Men	2,714	2.72%	96,957	97.28%
Women	5,835	4.78%	116,271	95.22%
Age [60,64)	150	0.31%	48,313	99.69%
Age [65,69)	291	0.65%	44,237	99.35%
Age [70,74)	633	1.55%	40,233	98.45%
Age [75,79)	1,503	4.35%	33,076	95.65%
Age [80,84)	1,957	8.55%	20,929	91.45%
Age [85,89)	2,381	12.58%	16,547	87.42%
Age [90,94)	1,304	14.76%	7,533	85.24%
Age [95,100)	330	12.27%	2,360	87.73%
Low HI	5,858	4.87%	114,309	95.13%
High HI	2,649	2.66%	96,838	97.34%
CCI 0–1	5,809	2.90%	194,257	97.10%
CCI 2–3	1,672	10.60%	14,108	89.40%
CCI > 3	1,068	18.01%	4,863	81.99%

HI: Socioeconomic status; CCI: Charlson comorbidity index

In Table SM1 (Supplementary material), we present the risk of comorbidity measured by the CCI according to household income, age and sex obtained by the linear regression model. Male sex and low household income were associated with more comorbidities. The CCI value, as the dependent variable, increased with age (2% per year with CI 2% and 2%), decreased with female sex (13% with CI 12% and 14%), and increased with low household income (4% with CI 4% and 5%).

**Table 2:** Sociodemographic and clinical characteristics of the new cases of dementia diagnosis in the population over 60 from 1 January 2021 to 1 January 2022 (incidence)

	New cases	Population 01/01/2021	Deaths	Prevalent cases	Person-years	Incidence/1000
Total	1,332	215,000	2,480	7,525	205,569	6.48
Men	499	96,386	1,211	2,324	93,178	5.36
Women	833	118,614	1,269	5,201	112,936	7.38
Age [60,65)	27	50,296	122	150	50,072	0.54
Age [65,70)	55	42,832	154	257	42,471	1.30
Age [70,75)	153	40,319	220	623	39,510	3.87
Age [75,80)	318	32,054	253	1,318	30,451	10.44
Age [80,85)	340	22,503	387	1,805	20,335	16.72
Age [85,89)	298	17,415	605	2,112	14,852	20.07
Age [90,105)	141	9,548	738	1,260	7,849	17.97
Low HI	851	118,589	1,765	5,256	112,025	7.60
High HI	481	96,411	715	2,269	93,544	5.14
CCI 0–1	888	195,573	2,252	5,266	188,737	4.70
CCI 2–3	271	14,593	160	1,433	12,945	20.94
CCI > 3	173	4,834	68	826	3,888	44.50

HI: Household income; CCI: Charlson comorbidity index

As shown in Table 2, crude incidence followed the same pattern as prevalence with the two gradients by CCI level and age and higher figures for women and low- household income individuals. The analyses with Poisson models (Table 3 and Table SM2) demonstrated the lack of significance of differences by household income when the incidence rate ratios (IRRs) of dementia were adjusted by age and sex (Baseline model) and by age, sex and CCI (model adjusted by CCI). In the baseline model, female sex had a higher and statistically significant IRR (IRR: 1.15; CIs: 1.02; 1.28), while the IRR for low household income was not statistically significant (IRR: 0.94; CIs: 0.84; 1.06). Adjustment for the comorbidity index increased the excess risk associated with female sex to an IRR of 1.31. This is because low- household income individuals had more comorbidities, were older and were more likely to be women. Women were significantly associated with higher IRRs in all the models, and the specific IRR rose with adjustment because women presented fewer comorbidities and because the percentage with low household income was higher than it was for men. The goodness-of-fit measured by the AIC statistic improved in the models adjusted by CCI and with interaction. The interaction was only significant for women and the CCI 2–3 group, indicating that in that combined group, the IRR was especially high (IRR: 1.42; CIs: 1.07; 1.89).

**Table 3:** Incidence rate ratios (IRRs) of dementia obtained with Poisson models adjusted for Charlson comorbidity index

Y = Dementia incidence	Model adjusted by CCI		
	IRR	CIs	*p*
Sex woman	1.31	1.17; 1.47	< 0.001
Age[65;69)	2.3	1.47; 3.7	< 0.001
Age [70;74)	6.58	4.45; 10.11	< 0.001
Age [75;79)	16.61	11.43; 25.2	< 0.001
Age [80;84)	24.61	16.94; 37.33	< 0.001
Age [85;89)	27.74	19.03; 42.2	< 0.001
Age [90;105)	25.36	17.04; 39.2	< 0.001
Low HI	0.92	0.82; 1.03	0.163
CCI 2–3	3.22	2.8; 3.68	< 0.001
CCI > 3	5.41	4.57; 6.37	< 0.001
AIC		432.68	

CCI: Charlson comorbidity index; IRR: Incidence rate ratios; CIs: Confidence intervals; HI: Household income; AIC: Akaike information criterion

**Fig. 1:**
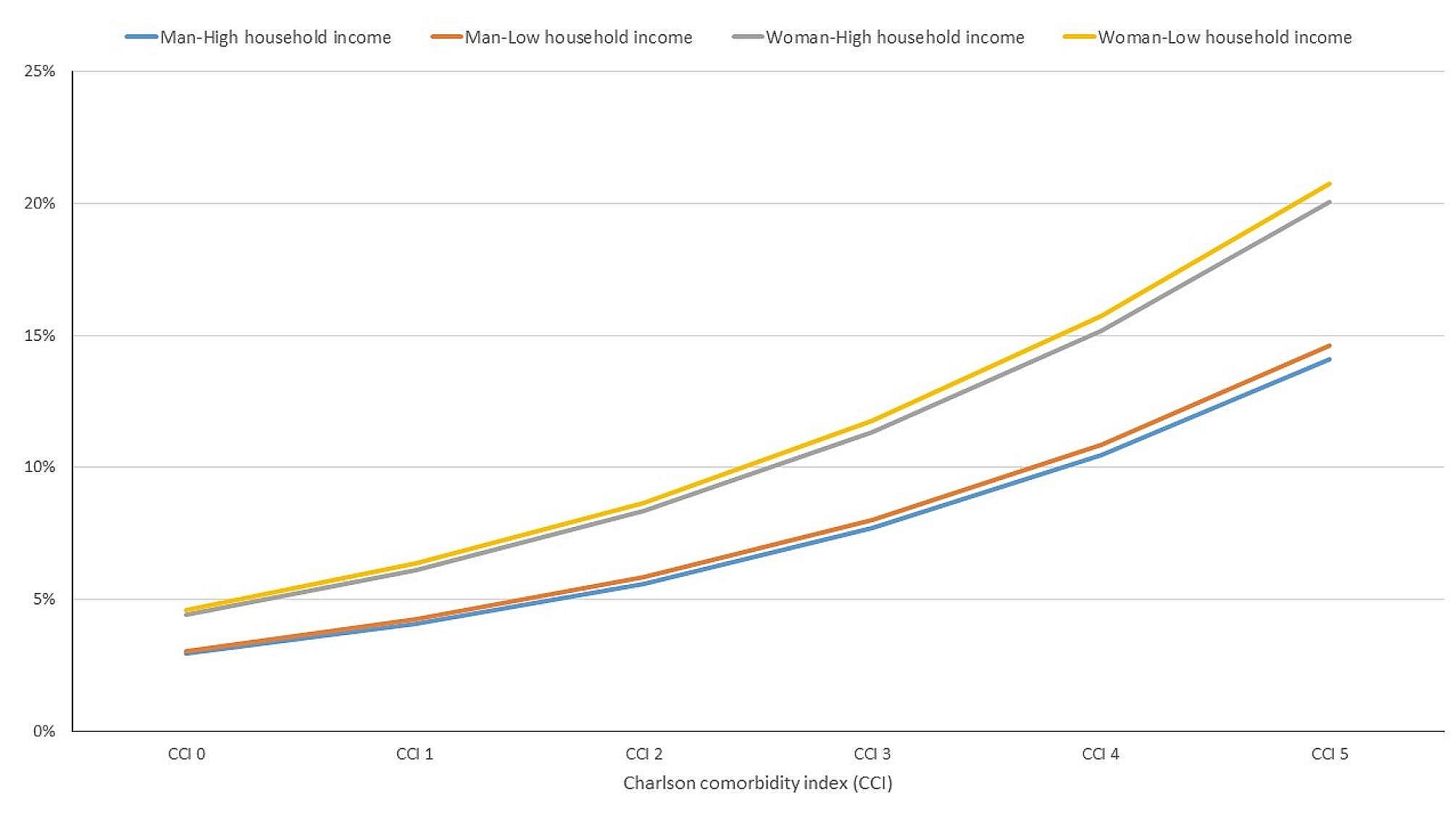
Likelihood of dementia for an 80-year-old person according to household income, sex and the CCI from 0 to 5 obtained with the logistic regression model

The logistic regression model that assessed dementia prevalence according to household income and sex and broken down by CCI and age as continuous covariates appears in Table SM2. The model from Table SM2 was used to calculate the likelihood of dementia for an 80-year-old according to household income, gender and CCI from 0 to 5 (Table SM3 and Fig. 1). The probability ranged from 5% for the CCI 0 group to 25% for the CCI 5 group. These results graphically display the key role of female gender and increasing CCI in the relationship between dementia prevalence and low household income. The lines by household income are close together, while the distance between the lines of men and women are very far apart.

Table 4 displays the logistic regression models with the prevalence of dementia as a dependent variable, sex, age and CCI as a binary covariate used in the mediation analysis. In the model adjusted for CCI, the ORs for female sex (OR = 1.44 with CI 1.32 and 1.57) and high CCI (OR = 3.36 with CI 3.09 and 3.65) showed a high risk of dementia, while the ORs for household income decreased and were no longer statistically significant (OR = 1.04 with CI 0.96 and 1.12). In Table 4, the interaction model shows that the risk of dementia associated to sex was not moderated by household income or CCI since the interactions were not statistically significant (*p* = 0.668 and *p* = 0.942).

**Table 4:** Logistic regression models used in the mediation analysis of dementia prevalence and low household income with female gender and the Charlson comorbidity index as binary covariates

	y = Dementia			
AUC: 0.828	OR	Upper CI	Lower CI	p
Low HI	1.04	1.12	0.96	0.381
CCI_>=2	3.36	3.65	3.09	< 0.001
Gender woman	1.44	1.57	1.32	< 0.001
Age	1.11	1.12	1.11	< 0.001
Woman*CCI_>=2	1.00	1.11	0.90	0.942
Woman*Low HI	1.02	1.13	0.92	0.668
	y = CCI (Binomial, >=2)			
AUC: 0.811	OR	Upper CI	Lower CI	p
Woman	0.68	0.70	0.66	< 0.001
Age	1.06	1.06	1.06	< 0.001
	y = HI (Binomial, low)			
AUC: 0.811	OR	Upper CI	Lower CI	p
Woman	1.80	1.83	1.77	< 0.001
Age	1.04	1.04	1.04	< 0.001

HI: Household income; CCI: Charlson comorbidity index. CI: confidence interval

The mediation analysis (Table 5), based on models from Table 4, estimated the percentage of the total relation between the exposure variable (sex) and the response variable (dementia) that is explained by the mediating variables (household income and CCI) meaning that they play a mediating role in the excess risk of dementia associated to female sex. All the indicators (Direct effect [OR: 2.04 with CIs: 1.97 and 2.10], Indirect effect [OR: 2.94 with CIs: 2.89 and 2.97], Total effect [OR: 2.14 with CIs: 2.07 and 2.20] and Explanation) were statistically significant. Both mediating variables explained jointly a 79% (CIs: 77% and 85%) of the response variable (dementia) associated to the exposure variable (female sex).

**Table 5:** Effect decomposition on the odds ratio of the dementia prevalence for women mediated by comorbidity and sex via the logistic regression-based approach

		Estimate	95% lower CI	95% upper CI	*P*
Rtnde	Direct effect	2.04	1.97	2.10	< 0.001
Rtnie	Indirect effect	2.94	2.89	2.97	< 0.001
Rte	Total effect	2.14	2.07	2.20	< 0.001
pm	Explanation	79%	77%	85%	< 0.001

CI: confidence interval

## Discussion

The main finding of this study is the mediating role of the growing comorbidity level and low household income on the increased risk of dementia associated with female sex. The CCI score and household income have opposite effects in the likelihood of dementia as the female sex is associated with greater comorbidity and is more likely than male to be in the low-income group. The reason for these different effects is that women tend to have fewer comorbidities and have longer life expectancies than men. Both variables showed in the descriptive analysis higher risk of dementia for women and people with low income. However, the mediation effect showed that the likelihood of dementia for women adjusted by CCI increased when compared to the baseline model. The risk was partially hidden by a lower rate of comorbidity. On the other hand, the higher risk of dementia in the group with lower household income was no longer statistically significant when the CCI and gender were included as covariates. The incidence risk analysis supports those findings, although the differences by income are not statistically significant in any of the Poisson models. In contrast, female sex is associated in a statistically significant manner with a higher incidence (IRR) in all models, which, similar to prevalence, increases when adjusting for the CCI.

Prevalence and incidence figures for women (OR: 1.44 and IRR: 1.31) indicating significant higher risk of dementia are similar to those of other observational studies based on electronic medical records, which also show a higher risk in women in the Catalan population, although they are somewhat lower in the Dutch population [2, 25, 43]. As Huque et al. note, sex differences in the prevalence of dementia can be explained by a longer life expectancy and social differences by gender, with women having fewer years of education and lower employment rates [9]. As expected, community studies using door-to-door surveys that identify both diagnosed and undiagnosed patients obtain higher incidence and prevalence figures for dementia [10, 13]. In the literature, there is no consensus about equality in the incidence of dementia by gender, but the prevalence is systematically higher in women, and variability by gender is explained by the higher life expectancy of women and their fewer years of education [8, 9]. In the same vein, work with high job control acts differently on the effect of the APOE ɛ4 allele in dementia for men and women by reducing the associated risk in men while producing an opposite pattern in women [18]. The estimated incidence is also similar to that described in the literature and, consistent with RWD studies, is higher in women [2, 43, 44]. There is no agreement in the literature about significant gender differences in dementia risk. Our figures showing a higher prevalence in women are in line with the majority of studies [8–12]. Ferreti et al. underscored the role of sex-specific patterns to be taken into account in the development of precision medicine for Alzheimer’s disease [36]. In the same line, genomic data have shown that the menopausal loss of oestrogen could underlie the increased risk [45]. Nevertheless, evidence about incidence rates by gender provides examples in both senses: some support an increased risk in women, while others do not show significant differences. Our finding of a higher IRR in women is consistent with the results of other studies that collected data from administrative registries [25, 43, 44]. In contrast, two reviews of the literature that included community studies did not find significant differences in incidence rates by sex [9, 46]. A possible explanation could be that women seek health care more frequently than men when they present memory complaints or other subjective cognitive symptoms. Therefore, they are identified and recorded within the health system. However, this justification does not fit well with the fact that male records contain more comorbidities.

Our finding of the mediating role of comorbidity in the risk of dementia does not correspond to other works that have measured inequalities in health. The use of healthcare resources and the risk of diabetes in the lower- household income population are higher despite adjusting for comorbidities [47, 48]. The reduction of inequalities by household income can be assessed through better wealth distribution, which is a social objective that is outside the scope of healthcare systems. To facilitate research on the greater impact of inequality, it has been suggested that epidemiological results should be presented in population terms and in a format that facilitates the involvement of policy-makers in this objective [49]. Strict control of comorbidities appears as a clinical implication of the excessive risk of dementia associated with a higher CCI (prevalence OR: 3.36 and IRR: 3.22) since reducing comorbidities also slows cognitive deterioration. The interpretation of the mediating role of comorbidity also allows the reduction of inequalities to be addressed through the control of comorbidities. The effect on dementia would be an added benefit since the efficiency of interventions to control comorbidities such as diabetes mellitus, stroke or ischaemic heart disease is measured without including the effects on dementia [49].

Dementia strikes different population groups unequally. Twelve risk factors have been identified, of which only 2 are included in the comorbidity index [35, 49]. Addressing the reduction of inequalities involves identifying the mediating mechanisms of the excess risk of the most disadvantaged groups. It is debatable whether the prevention approach should be conducted at the population or individual level [49]. Improving the educational level of the population and reducing social isolation and environmental pollution are population objectives that, if achieved, would also have the positive side effect of a reduction in dementia. Nevertheless, decision-making regarding these issues is outside the scope of the health system. The CCI shows high sensitivity to labelling comorbidities that appear in the life trajectories of individuals and that increase their risk of dementia. These health trajectories also depend on individuals’ socioeconomic circumstances and genetic burden [14, 15]. Given the inequality in the prevalence of comorbidities by household income, reducing or better controlling hypertension, diabetes, depression and obesity would not only prevent dementia but also reduce inequalities since absolute risk is higher in the most disadvantaged population. Thus, individual interventions to reduce comorbidities would also have a preventive effect on dementia. Unfortunately, despite have greater biological capacity to benefit from these interventions, individuals with lower income find harder to engage with them.

### Strengths and limitations

The strengths of this study were the large sample size, representing the epidemiology of dementia in a population of 221,777 individuals older than 60 years, and the availability of individual-level data for all the variables. While other studies have used county-level factors for socioeconomic indicators [9], household income for pharmaceutical copayment [27, 28] is an individual-level variable. However, the complex and interconnected nature of gender and income means biases in using household income that difficult to disentangle mediation effects.

The limitations of our study were its observational design, which did not allow for the demonstration of causality, and the lack of other determinants, such as genetics or education level. However, using designs other than RCTs allows for the use of population registers to demonstrate the changes required to implement policies at the population level for dementia risk reduction [49]. Given the strong modifying effect of education level on dementia, the analysis would have been more complete if it had included it as a social determinant that could reduce dementia risk by increasing cognitive reserve [50]. Since cognitive reserve delay the onset of dementia, educational level may also act as a mediating mechanism between household income and dementia risk, which has not been addressed in our work [51]. Using different dimensions of household income would be helpful to address the role of each mediating mechanism in the complex relationship between social determinants and dementia risk [52]. However, as a systematic review of articles about the causes of health inequality pointed out, only some studies have included third-factor explanations [22]. The intertwined relationship of comorbidity, household income, education, and gender as protective and risk factors for dementia could be assessed only partially in this study. Incorporating education would have improved the explanatory level of the model.

## Conclusions

In conclusion, we note that the level of comorbidity and household income act as mediators of the increased risk of dementia associated with gender. In addition, given the difference in the prevalence of comorbidities by household income, individual interventions to control them could not only prevent dementia but also reduce inequalities since the risk is higher in the most disadvantaged population.

### Electronic supplementary material

Below is the link to the electronic supplementary material.

Supplementary Material 1

## Data Availability

The data that support the findings of this study are available from [third party name] but restrictions apply to the availability of these data, which were used under license for the current study, and so are not publicly available. Data are however available from the authors upon reasonable request and with permission of the Basque Health Service (Email address: DIRECTORA.GENERALLISTA@osakidetza.eus).

## Declarations

### Authors’ information

The group of authors is made up of epidemiologists, clinicians, and computer engineers with complementary profiles.
