# Supplementary material for: Comorbidity and household income as mediators of gender inequalities in dementia risk: a real-world data population study
Source: BMC Geriatr. 2024 Feb 29;24:209. doi: 10.1186/s12877-024-04770-3 (PMC10905946; doi:10.1186/s12877-024-04770-3)
Supplement: Supplementary file 1 — Supplementary Material 1 [file 12877_2024_4770_MOESM1_ESM.pdf]

Supplementary material

Table SM1. Risk of co-morbidity measured by the Charlson comorbidity index according to household income and sex obtained by linear regression.

		Lower CI	Upper CI	p
Intercept	0.30	0.29	0.31	<0.001
Sex woman	0.87	0.86	0.88	<0.001
Age	1.02	1.02	1.02	<0.001
Low HI	1.04	1.04	1.05	<0.001

HI: household income; CCI: Charlson comorbidity index.

Table SM2. Incidence rate ratios (IRRs) of dementia obtained with Poisson models (baseline, adjusted for CCI, adjusted for CCI with SES*CCI interaction and adjusted for CCI with CCI*Sex interaction).

Y=Dementia incidence	Baseline Model			Model adjusted by CCI			Model adjusted by CCI with interaction			Model adjusted by CCI with interaction		
	IRR	CIs	p	IRR	CIs	p	IRR	CIs	p	IRR	CIs	p
Sex woman	1.15	1.02; 1.28	<0.05	1.31	1.17; 1.47	<0.001	1.31	1.17; 1.47	<0.001	1.22	1.06; 1.4	<0.01
Age[65;69)	2.39	1.52; 3.85	<0.001	2.3	1.47; 3.7	<0.001	2.3	1.47; 3.7	<0.001	2.31	1.47; 3.71	<0.001
Age [70;74)	7.16	4.84; 11.01	<0.001	6.58	4.45; 10.11	<0.001	6.59	4.45; 10.13	<0.001	6.61	4.47; 10.17	<0.001
Age [75;79)	19.26	13.26; 29.21	<0.001	16.61	11.43; 25.2	<0.001	16.68	11.48; 25.31	<0.001	16.72	11.51; 25.37	<0.001
Age [80;84)	30.77	21.2; 46.64	<0.001	24.61	16.94; 37.33	<0.001	24.73	17.02; 37.53	<0.001	24.77	17.05; 37.59	<0.001
Age [85;89)	36.73	25.24; 55.81	<0.001	27.74	19.03; 42.2	<0.001	27.87	19.12; 42.41	<0.001	27.91	19.14; 42.46	<0.001
Age [90;105)	32.69	21.99; 50.48	<0.001	25.36	17.04; 39.2	<0.001	25.47	17.11; 39.38	<0.001	25.47	17.11; 39.38	<0.001
Low SES	0.94	0.84; 1.06	0.318	0.92	0.82; 1.03	0.163	0.9	0.78; 1.03	0.124	0.92	0.82; 1.03	0.158
CCI 2-3				3.22	2.8; 3.68	<0.001	3.19	2.52; 4	<0.001	2.58	2.04; 3.24	<0.001
CCI>3				5.41	4.57; 6.37	<0.001	4.74	3.49; 6.3	<0.001	5.31	4.18; 6.68	<0.001
Low SES*CCI 2-3							1.01	0.76; 1.35	0.933			
Low SES*CCI>3							1.22	0.86; 1.74	0.276			
Woman*CCI 2-3										1.42	1.07; 1.89	<0.05
Woman*CCI>3										1.01	0.73; 1.4	0.947
AIC	880.92			432.68			435.46			430.72		

CCI: Charlson comorbidity index; IRR: Incidence rate ratios; CIs: Confidence intervals; SES: Socioeconomic status.

Table SM3. Likelihood of dementia obtained with logistic regression models using CCI and age as continuous variables

	Y=Dementia prevalence		
	OR	CIs	p
Sex women	1.53	1.46; 1.61	<0.001
Age	1.11	1.11; 1.11	<0.001
Low HI	1.04	0.99; 1.1	0.103
CCI	1.40	1.39; 1.42	<0.001

OR: Odds Ratio; HI: Household income; CCI: Charlson comorbidity index.

Table SM4. Probability of dementia for an 80-year-old according to SES, gender and CCI from 0 to 5 calculated with model from Table MS2.

		CCI 0	CCI 1	CCI 2	CCI 3	CCI 4	CCI 5
Sex	SES	P(Y|**X**)	P(Y|**X**)	P(Y|**X**)	P(Y|**X**)	P(Y|**X**)	P(Y|**X**)
Man	High	2.93%	4.06%	5.60%	7.69%	10.46%	14.08%
Man	Low	3.05%	4.23%	5.83%	7.99%	10.86%	14.60%
Woman	High	4.42%	6.09%	8.34%	11.32%	15.19%	20.09%
Woman	Low	4.60%	6.34%	8.67%	11.76%	15.75%	20.78%

SES: Socioeconomic status; CCI: Charlson comorbidity index.
